# Mechanical Properties of Novel 3D-Printed Restorative Materials for Definitive Dental Applications

**DOI:** 10.3390/ma18204662

**Published:** 2025-10-10

**Authors:** Moritz Hoffmann, Andrea Coldea, Bogna Stawarczyk

**Affiliations:** Department of Prosthetic Dentistry, University Hospital, LMU Munich, 80336 Munich, Germany; moritz.hoffmann@med.uni-muenchen.de (M.H.); andrea.coldea@med.uni-muenchen.de (A.C.)

**Keywords:** 3D printing, permanent restorations, resin-based printing, vat photopolymerization

## Abstract

The aim of this study is to evaluate the mechanical properties and long-term stability of 3D-printable resins for permanent fixed dental prostheses (FDPs), focusing on whether material performance is influenced by 3D-printer type or by differences in resin formulations. Specimens (N = 621) were printed. CAD/CAM blocks (BRILLIANT Crios) served as control. Flexural strength (FS) with elastic modulus (E_calc), Weibull modulus (m), Martens’ hardness (HM), indentation modulus (EIT), elastic modulus (E_RFDA), shear modulus (G_RFDA), and Poisson’s Ratio (ν) were measured initially, after water storage (24 h, 37 °C), and after thermocycling (5–55 °C, 10,000×). SEM analysis assessed microstructure. Data were analyzed using Kolmogorov–Smirnov, ANOVA with Scheffe post hoc, Kruskal–Wallis with Mann–Whitney U, and Weibull statistics with maximum likelihood (α = 0.05). A ceramic crown printed with Midas showed higher FS, HM, and EIT values after thermocycling than with Pro55s, and higher E_calc scores across all aging regimes. A Varseo Smile Crown Plus printed with VarseoXS and AsigaMax showed a higher FS value than TrixPrint2, while AsigaMax achieved the highest initial E_calc and E_RFDA values, and VarseoXS did so after thermocycling. HM, EIT, and G_RFDA were higher for TrixPrint2 and AsigaMax printed specimens, while ν varied by system and aging. 3Delta Crown, printed with AsigaMax, showed the highest FS, E_calc, HM, EIT, and m values after aging. VarseoSmile triniQ and Bridgetec showed the highest E_RFDA and G_RFDA values depending on aging, and Varseo Smile Crown Plus exhibited higher ν initially and post-aging. Printer system and resin formulation significantly influence the mechanical and aging behaviors of 3D-printed FDP materials, underscoring the importance of informed material and printer selection to ensure long-term clinical success.

## 1. Introduction

The continuous advancement of 3D-printing technologies has transformed workflows for manufacturing dental restorations. Progress in both printer systems and materials has expanded the use of polymer-based 3D-printing from basic auxiliaries, such as impression trays, to complex applications like surgical guides, denture bases, provisional restorations, and even permanent fixed dental prostheses (FDPs) [1]. Among available technologies, vat photopolymerization—particularly digital light processing (DLP)—has become highly relevant in dentistry due to its versatility, customizability, and short processing time [2].

Beyond the resin composition, key steps such as printing, washing, and post-polymerization critically affect the final properties of 3D-printed restorations [2,3]. Dental resins require precise control of exposure time, intensity, and wavelength to ensure adequate layer polymerization and minimize defects. Typical wavelengths range from 385 to 405 nm [4], directly influencing polymerization depth, mechanical performance, and surface quality [4,5,6]. Additional strategies, such as mixed-wavelength exposure, temperature-controlled resin vats, or pressure-assisted systems like digital press stereolithography (DPS), have been introduced to improve polymerization efficiency, dimensional accuracy, and printability—even for high-viscosity resins [7,8].

The washing process removes uncured resin from printed surfaces. Incomplete resin removal or inadequate washing can reduce the degree of conversion [9,10], potentially compromising biocompatibility and increasing the risk of adverse patient reactions [11]. Overexposure to solvents like isopropanol (IPA) can degrade surfaces [12,13,14], and ultrasonic baths increase evaporation and explosion hazards [15]. Alternative approaches, such as multi-stage washing or non-solvent techniques like centrifugation, aim to balance safety with effective resin removal [13].

Post-polymerization is equally critical. Parameters such as wavelength, intensity, and exposure time must be controlled, as residual monomers can impair mechanical performance and biocompatibility [16,17,18,19,20,21]. Thermal protocols can improve conversion rate and, in turn, mechanical strength and long-term stability [18,22], but over-curing risks material damage [15,22]. Using an inert gas atmosphere during post-curing can further improve surface quality by reducing oxygen inhibition [23,24].

Both closed and open system workflows are commercially available. Closed systems integrate printer, washing, and post-polymerization units with pre-set parameters for specific resins, ensuring process consistency. Open systems offer greater flexibility but introduce variability, especially in post-processing. Although general manufacturers guidelines exist, optimal settings for every device–resin combination cannot be standardized. Studies show that even with the same printer, resin choice strongly affects mechanical and esthetic outcomes, and post-processing strategies—particularly washing and post-polymerization—further influence performance [25]. A systematic review confirmed the pivotal role of the printer itself in determining final materials properties [26].

Most dental 3D-printing resins for permanent FDPs are based on methacrylate or/and acrylate monomers combined with highly reactive photoinitiators [1,12]. Ceramic fillers are added to enhance mechanical properties [27], but they also increase viscosity, which can impair flow, printability, and resolution [4,27]. Fillers can scatter light or cause refractive mismatches, reducing polymerization depth [28] and interlayer bonding, which ultimately compromises mechanical integrity and dimensional accuracy [27,29,30]. Three-dimensional-printing resins are relatively new, and only a few studies have investigated their long-term performance [8,31,32]. Early research suggests mechanical degradation under simulated aging, such as thermocycling [33,34]. In vitro protocols like cycles between 5 °C and 55 °C simulate the thermal stresses from daily food and drink intake [35,36,37]. Early reports already document decreases in flexural strength and elastic modulus, highlighting the need for further investigation.

Elastic properties can be assessed using various methods, including flexural testing (elastic modulus), instrumented indentation (indentation modulus), or resonance frequency damping analysis (RFDA) to determine dynamic elastic behavior, which—despite differences in principles—often reveal comparable trends [38,39].

Although 3D-printed resins show increasing potential in prosthetic dentistry, data on the long-term performance of newly developed materials for permanent restorations remain scarce, and the impact of different printing systems on their properties is largely unknown. To address these gaps, the present study systematically evaluated the mechanical and aging behavior of contemporary 3D-printing resins, from which the following central questions and null hypotheses were derived: (i) Do identical resin formulations exhibit differences in mechanical performance when printed using different 3D-printer systems? (ii) Do distinct resin formulations differ in their mechanical behavior when printed on the same printer under controlled conditions? The chosen materials represent contemporary 3D-printable resins intended for permanent prosthetic restorations, while the selected printers reflect currently available systems with varying printing technologies. This selection contributes to the systematic investigation of whether the same resin exhibits differences in mechanical or aging-related properties across different printing platforms. To address this, the following null hypotheses were formulated: (1) The ceramic crown resin (CCR) shows no differences in tested parameters when printed using a Midas printer versus a Pro 55s printer. The artificial aging shows no effect on the values. (2) The VarseoSmile Crown Plus resin (VSC) shows no differences in the tested parameters when printed using the Asiga Max printer, TrixPrint2 printer, or Varseo XS printer. The artificial aging shows no effect on the values. (3) Five resins—3Delta Crown (DCR), Bridgetec (BRT), Crowntec (CRT), Freeprint Crown (FCR), and VarseoSmile triniQ (VST)—printed with the Asiga Max printer do not differ in the tested parameters. The artificial aging shows no effect on the values. (4) The tested parameters of 3D-printed resins are comparable to those of milled block BRILLIANT Crios (BRC). Artificial aging shows no effect on the values. The tested parameters are flexural strength (FS) with elastic modulus (E_calc), Weibull modulus (m), Martens’ hardness (HM) with indentation modulus (EIT) and the outcomes from resonance frequency damping analysis elastic modulus (E_RFDA), shear modulus (G_RFDA), and Poisson’s Ratio (ν). An additionally hypothesis was built: (5) The method used to assess the elastic properties (E and related parameters like EIT)—whether by instrumented indentation, flexural testing, or RFDA—does not influence the observed trends between tested groups.

## 2. Materials and Methods

### 2.1. Specimen Fabrication

**Figure 1:**
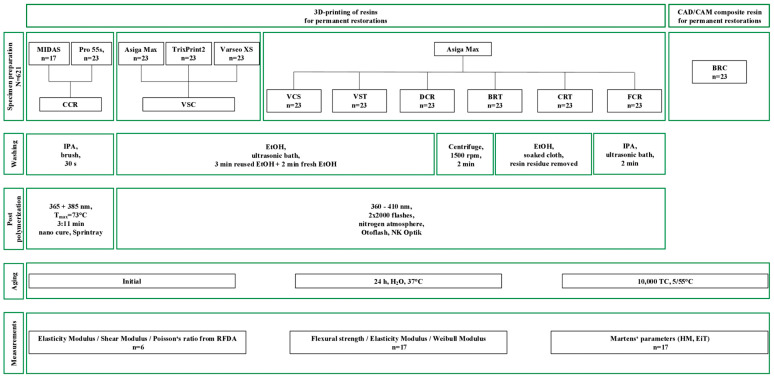
Study design.

Bar-shaped specimens were designed with the geometries I (1.2 × 4.0 × 12.0 mm) for destructive testing and II (7.9 × 30.6 × 70.0 mm) for nondestructive characterization (Rhino 7, Robert McNeel & Associates, Seattle, WA, USA). STL files were imported into the respective printer software, and specimens were oriented vertically at a 90° angle to the build platform. In total, N = 561 specimens (n = 17) were fabricated in geometry I, and N = 60 specimens (n = 6) in geometry II, using seven different 3D-printable resins: CCR, VSC, DCR, BRT, CRT, FCR, and VST (Figure 1). For geometry II, printing using the Midas was not possible due to size limitations.

The following five dental resin 3D-printers were used:

I. Midas, technology: DPS with λ = 385 nm, Sprintray Inc., Los Angeles, CA, USA;
II. Pro 55s, technology: DLP with λ = 405 nm, Sprintray Inc., Los Angeles, CA, USA;
III. Asiga Max, technology: DLP with λ = 385 nm, ASIGA, Sydney, Australia;
IV. TrixPrint2, technology: DLP with λ = 385 nm, DEKEMA, Freilassing, Germany;
V. Varseo XS, technology: DLP with λ = 405 nm, BEGO Bremer Goldschlägerei Wilh. Herbst, Bremen, Germany.

Printing was performed with a layer resolution set to 50 µm across all prints, except for the Midas (100 µm due to system specifications). After printing, specimens were cleaned of residual resin, dried with oil-free air, and post-polymerized according to manufacturer specifications (Table 1). Support structures were manually removed.

For the control group (CG), CAD/CAM resin composite blocks (BRILLIANT Crios, Coltène/Whaledent AG, Altstätten, Switzerland; LotNo.: N28617) were cut into bar-shaped specimens using a precision cutting machine (Secotom 50, Struers, Ballerup, Denmark) under water cooling (feel rate: 0.07 mm/min, speed: 3500 rpm) and finished with chamfered edges using P500 silicon carbide paper (Struers).

### 2.2. Artificial Aging

Geometry I specimens underwent three aging regimes to simulate 12 months of clinical service: (a) initial (post-manufacture), (b) for 24 h storage in deionized water at 37 °C, and (c) 10,000× thermocycles between 5 °C and 55 °C (30 s dwell time) [35].

### 2.3. Flexural Strength with Elastic Modulus

Three-point bending tests were performed (10 mm span, 1 mm/min crosshead speed) until fracture occurred using a universal testing device (RetroLine 1445, Zwick/Roell, Ulm, Germany). The FS was calculated using the following equation:

$$FS=\frac{3Fl}{2wh^{2}}$$

where FS: 3-point FS (MPa); F: fracture load (N); l: bearing distance (mm); w: specimen width (mm); and h: specimen height (mm).

E was calculated (E_calc) using the following equitation:

$$E=\frac{F_{1}l^{3}}{4wh^{3}d}÷1000$$

where E: elastic modulus (GPa); F_1_: load (N) at the end of the elastic region of the load-deflection curve; l: bearing distance (mm); w: specimen width (mm); h: specimen height (mm); and d: deflection at F_1_ (mm).

### 2.4. Martens’ Parameters

HM and EIT were determined using a universal hardness testing device (ZHU 0.2, Zwick/Roell). The bars were positioned on their wide side and subjected to a load of 9.81 N and 10 s dwell time with a maximum indentation depth below one-third of the specimen thickness, utilizing a Vickers diamond indenter with an angle of α = 136. The values for HM and EIT were calculated with the testing software (testX-pertV12.3, Master, Zwick/Roell) using the following equations:

$$HM=\frac{F}{A_{s}(h)}$$

where HM: Martens’ hardness (N/mm^2^); F: loading (N); and A_s_(h): penetrated area of the indenter at the distance h from tip to specimen surface (mm^2^).

$$E_{IT}=(1-v_{S}^{2}){\left(\left(2\sqrt{\frac{A_{P}(h_{c})}{\sqrt{\pi }S}}\right)-\left(\frac{1-v_{i}^{2}}{E_{i}}\right)\right)}^{-1}$$

where E_IT_: elastic indentation modulus (N/mm^2^); A_p_(h_c_): contact area at the loading (mm^2^); ν: Poisson ratio of specimen (ν_S_ = 0.35) and indenter (ν_i_ = 0.3); and S: contact stiffness obtained from force removal curve.

### 2.5. Resonant Frequency Damping Analysis (RFDA)

Specimens from geometry II were used for RFDA using an acoustic setup (RFDA system 20, IMCE, Genk, Belgium). Measurements were conducted at a controlled temperature of 21 °C, using an automatically excited stainless-steel profile with a thickness of 3 mm, in accordance with ASTM E1876-22 [40].

The E_RFDA, G_RFDA, and ν were calculated with the testing software (RFDA Essential v1.0.2, IMCE) using the following equations, as specified by ASTM E1876-22:

$$E=0.9465\left(\frac{mf_{f}^{2}}{w}\right)\left(\frac{L^{3}}{t^{3}}\right)T_{1}$$

where E: elastic modulus (Pa); m: mass of the bar (g); w: width of the bar (mm); L: length of the bar (mm); t: thickness of the bar (mm); f_f_: fundamental resonant frequency of the bar in flexure (Hz); and T_1_: correction factor for fundamental flexural mode.

$$G=\frac{4Lmf_{t}^{2}}{bt}\left[B/(1+A)\right]$$

where G: shear modulus (Pa); L: length of the bar (mm); m: mass of the bar (g); f_t_: fundamental resonant frequency of the bar in torsion (Hz); b: width of the rectangular cross-section (mm); t: thickness of the rectangular cross-section (mm); B: correction factor for torsion mode; and A: correction factor for geometry.

$$\nu =\left(\frac{E}{2G}\right)-1$$

where ν: Poisson’s ratio; E: elastic modulus (Pa); and G: shear modulus (Pa).

### 2.6. Microstructural Analysis

Microstructures of the specimens were analyzed by scanning electron microscopy (SEM) (Zeiss Supra 55 VP, Carl Zeiss, Oberkochen, Germany) at 5000× magnification. For particle size analysis, Fiji (ImageJ distribution, version.2.16.0) [41] was used to evaluate the Feret diameter. A minimum particle size threshold of 0.14 µm was applied.

### 2.7. Statistical Analysis

Descriptive statistics were calculated first. Kolmogorov–Smirnov tests were used to assess deviations from a normal distribution for each parameter within each group. Parameters with normally distributed data (FS, HM, EIT, E_calc) were analyzed using parametric tests—independent t-tests and one-way ANOVA followed by Scheffé’s post hoc test. Non-normally distributed data (E_RFDA, G_RFDA, ν) were analyzed using the Kruskal–Wallis test with Mann–Whitney U tests for post hoc comparisons. Statistical significance was set at α = 0.05. All statistical analyses were performed using SPSS v30.0 (IBM Corp., Armonk, NY, USA). The Weibull modulus was calculated via maximum likelihood estimation with 95% confidence intervals [42].

## 3. Results

**Figure 2:**
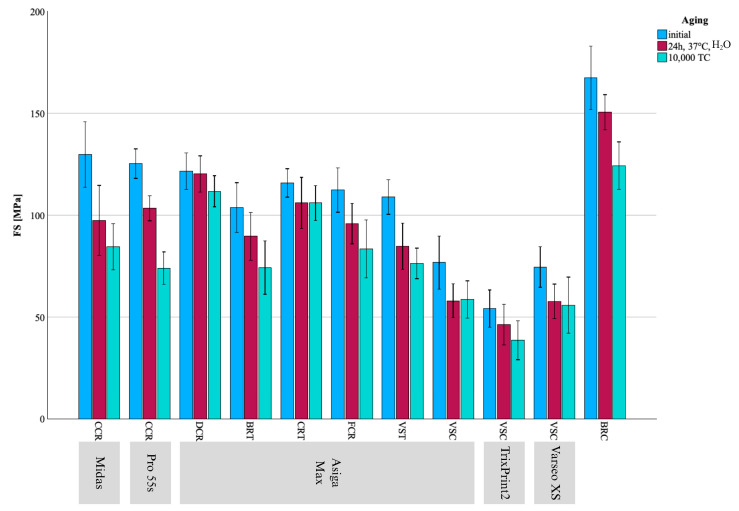
Mean ± SD for FS results presented as bar chart across all groups and aging regimes.

**Figure 3:**
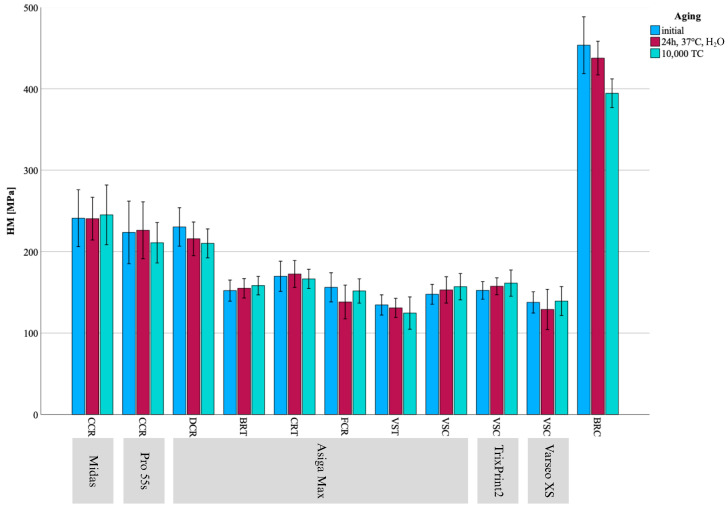
Mean ± SD for HM results presented as bar chart across all groups and aging regimes.

**Figure 4:**
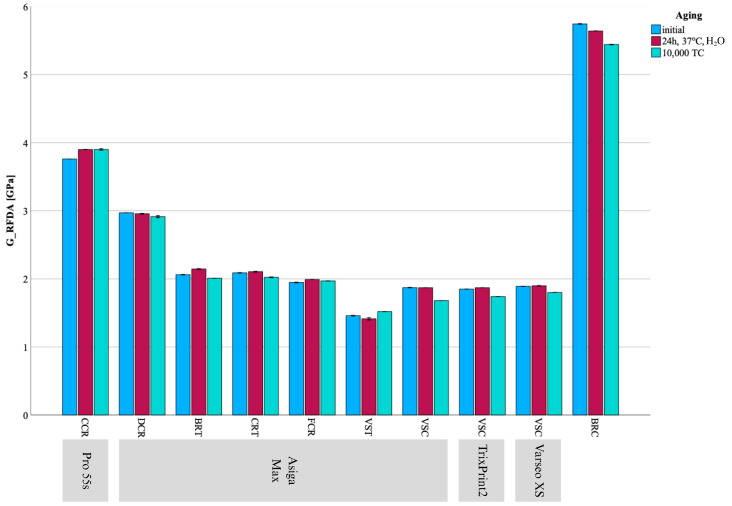
Mean ± SD for G_RFDA results presented as bar chart across all groups and aging regimes.

**Figure 5:**
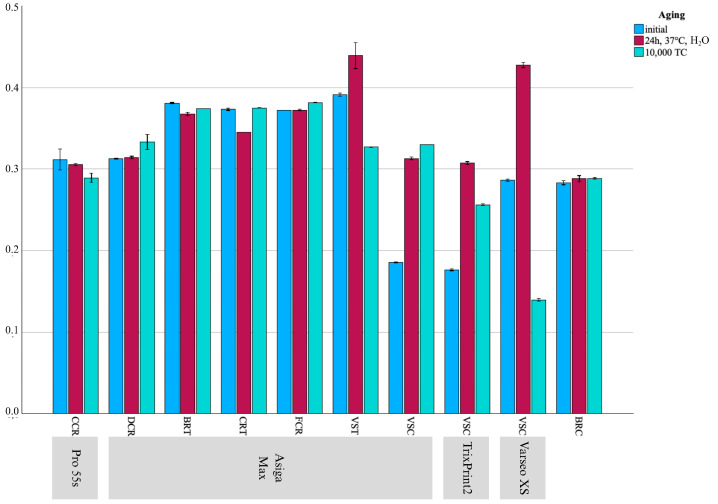
Mean ± SD for ν results presented as bar chart across all groups and aging regimes.

**Figure 6:**
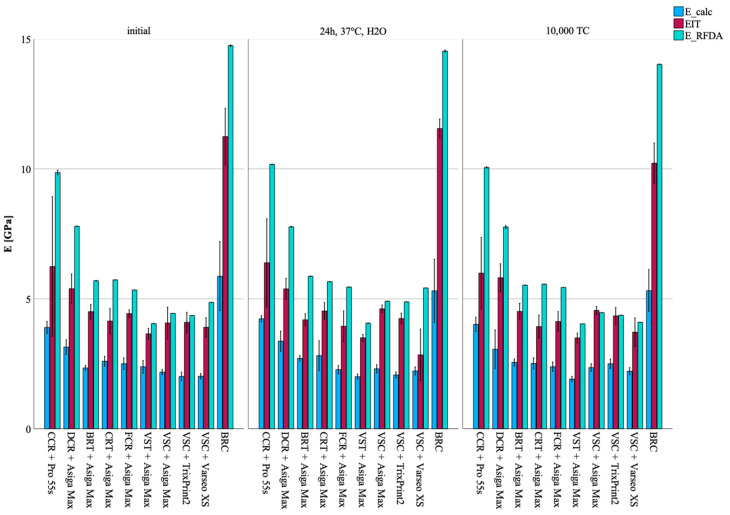
Mean ± SD for E_calc, EIT, E_RFDA, and ν results presented as bar charts across all groups and aging regimes.

HM, EIT, FS, and E_calc showed no deviation from normal distribution; therefore, parametric tests were used for data analyses. In contrast, E_RFDA, G_RFDA, and ν deviated from normal distribution, and nonparametric tests were used for analyses. All results are visualized in Figure 2, Figure 3, Figure 4, Figure 5 and Figure 6 and summarized in Table 2, Table 3 and Table 4.

### 3.1. Impact of Printer Within CCR

After thermocycling, specimens printed with Midas showed higher FS (*p* = 0.002), HM (*p* = 0.002), and EIT (*p* = 0.004) values compared to those printed with Pro 55s. Notably, this trend was also observed within EIT values after water storage (*p* = 0.039), as well as within E_calc across all aging regimes (*p* = 0.023–0.069). Within the Pro 55s group, the Weibull modulus was higher for the initial (m = 19.9) and after water storage groups (m = 20.7) compared to the thermocycled group (m = 10.7).

### 3.2. Impact of Printer Within VSC

VSC printed with Varseo XS and Asiga Max showed higher FS values compared to those printed with TrixPrint2 (*p* < 0.001). Regarding E_calc values, the Asiga Max group showed higher values (*p* < 0.001) than the Varseo XS and TrixPrint2 groups, whereas after water storage, Asiga Max maintained higher E_calc values compared to Varseo XS (*p* < 0.001). In contrast, after thermocycling, TrixPrint2 and Asiga Max led to higher E_calc values compared to Varseo XS (*p* < 0.001). TrixPrint2 and Asiga Max yielded higher HM (*p* = 0.003) and EIT (*p* < 0.001) values than Varseo XS. Regarding E_RFDA, Asiga Max produced the highest initial values (*p* = 0.001), whereas after water storage, TrixPrint2 led to the highest E_RFDA values (*p* = 0.001). Following thermocycling, Varseo XS showed higher E_RFDA values than the other printers (*p* = 0.001). For G_RFDA, TrixPrint2 showed higher initial values compared to Asiga Max and Varseo XS (*p* = 0.001). After water storage, TrixPrint2 and Asiga Max showed higher G_RFDA values than Varseo XS (*p* = 0.001), and after thermocycling, Asiga Max produced higher values than TrixPrint2 and Varseo XS (*p* = 0.001). Regarding ν, TrixPrint2 displayed the highest initial values and values after water storage compared to Asiga Max and Varseo XS (*p* = 0.001), whereas after thermocycling, Varseo XS showed the highest ν (*p* = 0.001).

### 3.3. Impact of the Resin Within the Asiga Max Printer

DCR demonstrated higher mechanical properties across most parameters. It showed higher FS values both initially (*p* < 0.001) and after water storage compared to all other groups (*p* < 0.001). Whereas after thermocycling, DCR and CRT showed the highest FS values (*p* < 0.001). For E_calc values, DCR printed with Asiga Max consistently exhibited the highest values across all aging regimes (*p* < 0.001). The Weibull modulus revealed initially higher reliability for CRT (m = 19.7), whereas after water storage and thermocycling, DCR presented the highest reliability (m = 15.8 and m = 18.0, respectively), with other groups showing considerably lower values (m = 6.4–8.7). Regarding HM and EIT, DCR consistently exhibited highest values across all aging regimes (*p* < 0.001), while CRT, FCR, and BRT showed comparable ranges. For E_RFDA, VST demonstrated the highest initial values and water-stored (*p* = 0.001), whereas BRT exhibited the highest after thermocycling (*p* = 0.001). G_RFDA was consistently higher for DCR under all aging regimes (*p* = 0.001). As for ν, VSC showed initially elevated values compared to DCR, CRT, and FCR (*p* < 0.001), followed by BRT and VST (*p* = 0.001). After water storage, DCR and VSC presented the highest values (*p* = 0.001), while after thermocycling, VST showed the highest, followed by DCR and VSC with BRT, CRT, and FCR showing lower values (*p* = 0.001).

### 3.4. Comparability to the CG

Across all aging regimes, CG consistently exhibited the highest values for E_calc, HM, EIT, E_RFDA, and G_RFDA (*p* < 0.001–0.004). CG demonstrated the highest FS both initially and after water storage compared to all other groups (*p* < 0.001). After thermocycling, CG remained within the same value range as DCR and CRT (*p* = 0.827–>0.999). The Weibull modulus showed higher reliability for CG (m = 12.3) compared to VSC printed with Asiga Max (m = 6.6). After water storage, CG (m = 20.2) maintained higher values compared to VSC printed with TrixPrint2 (m = 5.6), CCR printed with Midas (m = 6.8), VSC printed with Varseo XS (m = 7.8), VSC printed with Asiga Max (m = 8.3), VST (m = 8.4), BRT (m = 8.7), CRT (m = 9.4), and FCR (m = 11.2). After thermocycling, CG (m = 12.9) still exhibited higher values than VSC printed with TrixPrint2 (m = 5.0), VSC printed with Varseo XS (m = 5.6), BRT (m = 6.4), and FCR (m = 6.9). Regarding ν, CG showed higher values compared to VSC printed with Asiga Max (*p* = 0.002) or TrixPrint2 (*p* = 0.003), but lower values compared to CCR printed with Pro 55s (*p* = 0.003), as well as DCR (*p* = 0.003), BRT (*p* = 0.003), CRT (*p* = 0.003), FCR (*p* = 0.002), and VST printed with Asiga (*p* = 0.002), and VSC printed with Varseo XS (*p* = 0.003). After water storage, CG showed lower ν values than all tested groups (*p* = 0.003–0.004). Following thermocycling, CG exhibited the highest values compared to VSC printed with Varseo XS (*p* = 0.003), but the lowest compared to DCR (*p* = 0.004), BRT (*p* = 0.004), CRT (*p* = 0.003), FCR (*p* = 0.002), VST (*p* = 0.002), and VSC printed with Asiga Max (*p* = 0.002) or TrixPrint2 (*p* = 0.002).

### 3.5. Method to Assess the Elastic Properties

Across all groups, E values of initially tested specimen varied depending on the measurement method. While absolute values differed, consistent trends were observed: E_RFDA yielded higher values than EIT and E_calc. Furthermore, E_RFDA showed the lowest variability across all methods.

### 3.6. Results for Microstructural Analysis (Figure 7, Figure 8 and Figure 9)

CCR (A, B) exhibited irregular, sharp-edged filler particles with a broad particle size distribution, ranging from 0.14 to 8.77 µm with a filler content of 43 area% when printed with Midas, and 0.14–7.69 µm with a filler content of 33 area% when printed with Pro 55s. The particle distribution appeared more homogenous in CCR printed with Midas than with Pro 55s (A, B). VSC (C, D, E) predominantly showed rounded particles with narrow size distributions of 0.14–2.82 µm and a 20 area% filler content when printed with Asiga Max (C), 0.14–3.80 µm and a 26 area% with TrixPrint2 (D), and 0.14–2.87 µm and an 18 area% with Varseo XS (E). Notably, specimens printed with TrixPrint2 (D) exhibited more pores compared to those printed with Asiga Max (C) or Varseo XS (E). VST printed with Asiga Max (F) presented a mixture of angular and rounded particles, with a narrow particle size distribution of 0.14–2.79 µm and a filler content of 16 area%. DCR printed with Asiga Max (G) showed edged particles with a narrow distribution of 0.14–2.60 µm and 21 area% filler content, while BRT (H) and CRT (I) showed similar edged particles with distributions of 0.14–1.76 µm (18 area%) and 0.14–2.04 µm (13 area%), respectively. FCR printed with Asiga Max (J) exhibited edged particles with a broad particle size distribution of 0.14–5.71 µm and a filler content of 23 area%. Finally, BCR (K) showed a mixture of rounded and edged particles with a narrow distribution of 0.14–3.79 µm and a filler content of 39 area%.

## 4. Discussion

This study evaluated the influence of different 3D-printing systems and resin formulations, all processed under validated washing and post-polymerization protocols, on the mechanical properties and long-term stability of resins intended for permanent FDPs. Differences were observed between groups, depending on 3D-printer type, resin formulation, aging regimes, and in comparison to the CG. Furthermore, the trends seen in elastic properties were influenced by the specific testing method, leading to the rejection of all null hypotheses. By strictly adhering to manufacturers’ validated workflows—including predefined printing, washing, and post-polymerization parameters—the study aimed to replicate clinically relevant conditions while minimizing processing-related bias, enabling a focused evaluation of printer and material-specific properties.

### 4.1. Impact of Printer

Differences in mechanical outcomes between printers can largely be attributed to variations in printing technologies. This study included DPS (Midas) and DLP (Pro 55s, Asiga Max, TrixPrint2, Varseo XS) processing. DPS applies pressure during printing, which may help reduce microstructural inhomogeneities [8] and allows the use of higher-viscosity resins [7]. As a comparatively recent development, DPS has yet to be thoroughly investigated in the scientific domain. Layer thickness is a known factor influencing the mechanical properties—specimens printed at 50 µm generally exhibit superior properties compared to those printed at 100 µm [43]. Additionally, layer thickness is directly linked to the degree of conversion, affecting not only mechanical performance but also long-term stability and biocompatibility [21]. Interestingly, contrary to existing literature, this study observed FS differences between layer thicknesses only after thermocycling (10,000 cycles), suggesting that DPS technology may mitigate the negative effects of increased layer thickness—an intriguing hypothesis warranting further investigation.

The differences in E_calc observed between Midas and Pro 55s cannot be explained by layer thickness alone [43]. Notably, these two printers operate at different photopolymerization wavelengths (Midas: 385 nm; Pro 55s: 405 nm). Since the absorption spectra of photoinitiators such as trimethylbenzoyl diphenylphosphine oxide (TPO) are wavelength-dependent, variations in wavelength can influence the degree of conversion and thus mechanical properties [44]. Shorter wavelengths are typically absorbed more efficiently by TPO, potentially enhancing polymerization and yielding stiffer polymer networks [45]. Another contributing factor is the filler content. Assuming identical resin formulations, the printing process itself may impact the filler content in the final objects. Microstructural analysis of CCRs printed with Midas or Pro 55s revealed a 10% difference in filler content. Given that higher filler content is generally associated with increased elastic modulus [46], this may have contributed to the observed variations. Differences in m-values may also be linked to process-related variations affecting interlayer bonding quality [47]. Similar explanations apply to the observed differences in FS, E_calc, HM, and EIT among VSC specimens printed with Asiga Max, TrixPrint2, and Varseo XS. While Asiga Max and TrixPrint2 operate at λ = 385 nm, the Varseo XS operates at λ = 405 nm. Additionally, hardware-specific light intensities across printers affect polymerization efficiency and thus the mechanical performance of the printed FDPs [47,48].

### 4.2. Impact of the Resin

**Figure 7:**
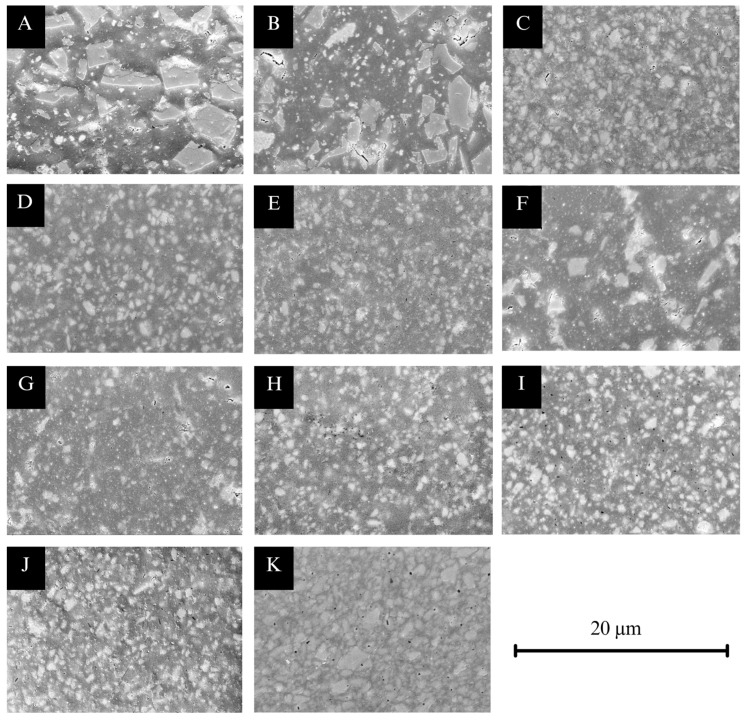
SEM micrographs (5000× magnification): (**A**) CCR + Midas, (**B**) CCR + Pro 55s, (**C**) DCR + Asiga Max, (**D**) BRT + Asiga Max, (**E**) CRT + Asiga Max, (**F**) FCR + Asiga Max, (**G**) VST + Asiga Max, (**H**) VSC + Asiga Max, (**I**) VSC + TrixPrint2, (**J**) VSC + Varseo XS, and (**K**) BCR.

Differences in mechanical performance among the tested resins are largely attributed to their specific formulations [49]. Incorporating fillers into resin formulations generally enhances mechanical properties [32]. However, increasing filler content also raises viscosity, potentially compromising printability [4,27]. The results of this study reflect this complexity. FCR, which had the highest area filler content, displayed structural flaws and porosities (Figure 7), likely resulting from limited printability [8] or insufficient filler–matrix adhesion. Filler-related factors, such as material type, particle shape, and particle size distribution, are known to influence both mechanical properties and printability [32]. SEM micrographs allowed the evaluation of particle shape and size: quantitative particle size distributions were not determined. A broad particle size distribution can improve packing density and reduce interstitial space, enhancing strength, while large agglomerate or poorly dispersed particles may weaken the material—yet these effects were not fully resolved in Weibull analysis in this investigation. The chemical composition of the resin-matrix monomers plays a crucial role in determining the polymer network architecture. Unfortunately, detailed information on monomer composition was not available for all tested resins. As discussed, the match between printer wavelength, light intensity, and the resin’s chemical composition—particularly the photoinitiator system—is essential to achieving optimal polymerization. Moreover, the resin-specific washing protocols can affect the mechanical performance. Recent studies showed that prolonged exposure to organic solvents such as ethanol or isopropanol can reduce FS, HM by causing swelling, filler leaching, and interference with post-curing. In contrast, non-solvent methods like centrifugation or shorter washing protocols have been reported to better preserve mechanical properties [9,10,13,14,21,28]. The results of this investigation align with aforementioned previous studies in which VSC and VST underwent 5 min ultrasonic solvent bath, FCR was washed for 2 min, BRT and CRT were cleaned with cloth, and DCR was processed using centrifugation—a non-solvent approach. Interestingly, DCR showed higher mechanical properties than FCR despite having lower filler content, suggesting that differences in washing protocols may partly explain these outcomes.

### 4.3. Comparability to CG

Differences in the performance of printed specimens compared to those of conventional CAD/CAM blocks can be attributed to differences in their formulation—most notably the filler content and the degree of polymer network crosslinking [8]. In the present investigation, CCR exhibited a slightly higher filler content (area%) than BRT, yet showed inferior mechanical properties in all tested parameters except for E_Calc. This discrepancy may be explained by differences in filler quantification methods; while CCR demonstrates a relatively high filler content in terms of area%, the literature indicates that its filler weight% at 50wt% [11,50] is lower compared to BRT at 70.7wt% [8]. This highlights that area-based filler analysis and gravimetric filler analysis are not directly comparable.

Another key difference is the industrial polymerization process for CAD/CAM resins, conducted under controlled conditions, which involves elevated temperatures and pressures [50] and results in a highly homogeneous and crosslinked polymer network [51]. This is also reflected in HM in this investigation and can be supported by other investigations [52].

### 4.4. Comparability of the Methods to Assess the Elastic Properties

Tendencies between the tested methods—modulus calculation from FS, EIT, and RFDA—across all groups remained consistent. Systematic differences in absolute values are well documented in the literature [38]. The methods used are based on distinct loading principles, such as bending, local surface indentation, and dynamic vibration. FS testing reflects global stiffness and is more sensitive to defects and porosity, while EIT reflects surface properties and is strongly affected by local deformation behavior [38]. RFDA measures the bulk elasticity and is characterized by low susceptibility to volume or surface defects and has a high producibility [38]. Despite the different physical principals of the methods, comparable trends have been reported in previous studies on resin-based composites or zirconia. The findings of this investigation confirm the robustness of the intergroup comparisons of the observed elastic properties. Observations can be explained by the method-specific susceptibility to errors. E_calc might be highly affected by volumetric flaws (e.g., porosities from printing process), and EIT by local inhomogeneities between matrix and filler particles [38]. Further differences can be attributed to the derivation of E_calc from FS and the corresponding strain, which represents an engineered approximation rather than a standardized flexural modulus [38,53].

### 4.5. Limitations and Future Recommendations

The layer resolution was set to 50 µm across all prints, except Midas where 100 µm was used due to system specifications—this aspect should be considered. Nevertheless, the concept of this investigation was based on using the recommended manufacturing parameters for permanent FDPs, and the present layer resolutions were applied. For future studies, however, it would be of interest to evaluate the influence of layer resolution on the mechanical parameters. To achieve this, it would be essential to correctly adjust the printing parameters (e.g., polymerization exposure of each layer) to avoid over- or under-polymerization.

Another limitation lies in the different cleaning protocols used, which in the present study design also followed the manufacturers’ recommendations. For future investigations, it would be advisable to evaluate the effect of different cleaning protocols across groups in order to distinguish the potential effects of the resin formulations from those of the post-processing methods.

Detailed analyses of the chemical composition of the exact filler formulations of the used resins were not performed. While SEM micrographs allowed for an evaluation of particle size and morphology, no quantitative particle size distribution was performed. As a limitation, it must be noted that area-based and gravimetric filler analyses are not directly comparable, as filler density, particle size distribution, and particle orientation play a decisive role. Moreover, a comprehensive chemical characterization would provide deeper insights into the differences between the investigated resins—future studies should therefore address these aspects.

## 5. Conclusions

The mechanical performance of 3D-printed FDPs for permanent restorations is affected by both the printing system and the resin formulation. Even when the same resin was used, FDPs printed on different printers exhibited varying mechanical properties. These differences were evident not only between printing technologies (E_DPS_ > E_DLP_) but also among printers using the same technology (e.g., VSC with DLP; FS_AsigaMax_, FS_VarseoXS_ > FS_TrixPrint2_), underlining the relevance of validated user workflows to achieve reproducible results.

CAD/CAM materials showed superior mechanical properties, but not necessarily greater aging resistance. For clinical application, the anticipated functional load and the specific indication should therefore guide the choice of both technology and material. This selection should also consider the process-related advantages and disadvantages of each system, as these factors may critically affect long-term performance.

The method used to determinate E should match specimen and material conditions, although general trends remained consistent across tested methods.

## Figures and Tables

**Figure 8:**
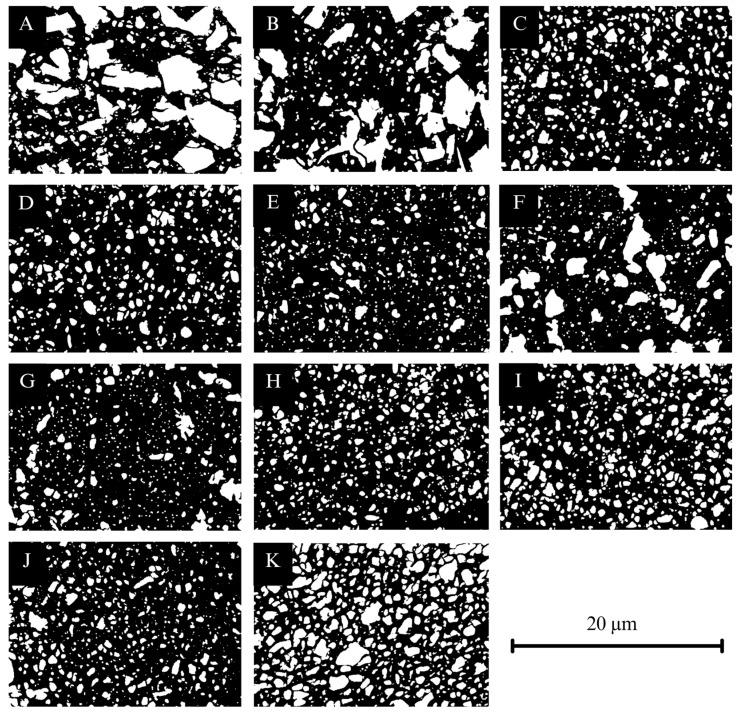
Thresholded SEM micrographs (5000× magnification) for particle size analysis: (**A**) CCR + Midas, (**B**) CCR + Pro 55s, (**C**) DCR + Asiga Max, (**D**) BRT + Asiga Max, (**E**) CRT + Asiga Max, (**F**) FCR + Asiga Max, (**G**) VST + Asiga Max, (**H**) VSC + Asiga Max, (**I**) VSC + TrixPrint2, (**J**) VSC + Varseo XS, and (**K**) BCR.

**Figure 9:**
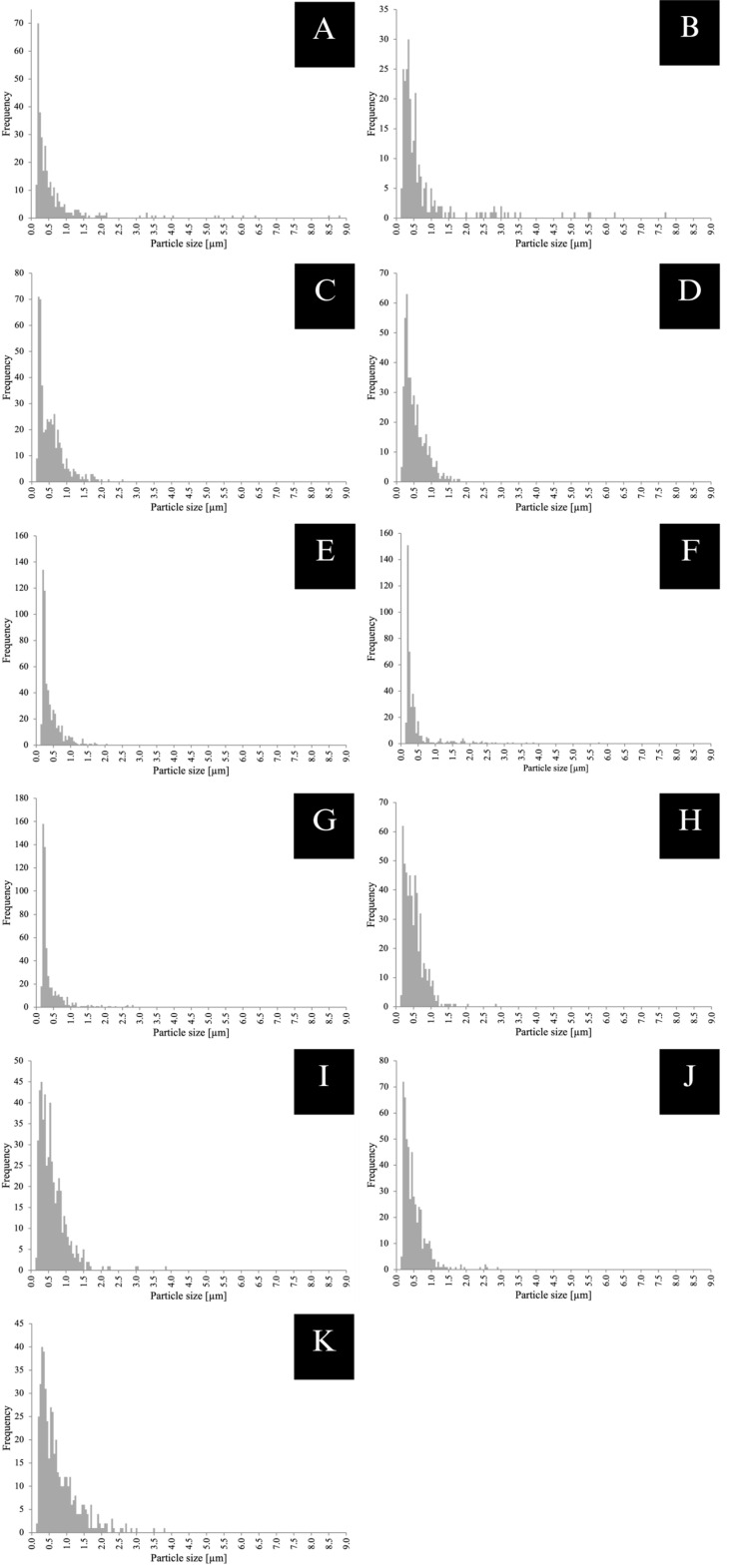
Particle size distribution of (**A**) CCR + Midas, (**B**) CCR + Pro 55s, (**C**) DCR + Asiga Max, (**D**) BRT + Asiga Max, (**E**) CRT + Asiga Max, (**F**) FCR + Asiga Max, (**G**) VST + Asiga Max, (**H**) VSC + Asiga Max, (**I**) VSC + TrixPrint2, (**J**) VSC + Varseo XS, and (**K**) BCR.

**Table 1:** Performed resin specific cleaning and post-polymerization protocol.

Resin	Abbreviation	Manufacturer	Lot No.	Cleaning Protocol	Post-Polymerization Protocol
Ceramic Crown	CCR	SprintRay Inc., Los Angeles, CA, USA	M24K008 (Midas) S24D012 (Pro 55s)	Brush + isopropyl alcohol (99.7%. SAV Liquid ProductionFlintsbach am Inn, Germany), 30 s. Dried with an air syringe.	Pro Cure (SprintRay), 365 + 385 nm. Ceramic Crown curing program. Normal mode
3Delta Crown	DCR	DeltaMed, Friedberg, Germany	240403A	Centrifugation (Multifuge 1 S-R; Heraeus. Hanau, Germany), 1500 rpm, 2 min, and 20 °C.	Otoflash G171 (NK Optik. Baierbrunn. Germany). 12.5 mW/cm^2^ per flash 360–410 nm, 2 × 2000 flashes. Nitrogen atmosphere. A total of 2000 flashes each side.
Bridgetec	BRT	Saremco Dental, Rebstein, Switzerland	280824-01	Ethanol-soaked cloth (96%. Otto Fischar. Saarbrücken, Germany). Dried with an air syringe.	
Crowntec	CRT		F493		
Freeprint Crown	FCR	DETAX, Ettlingen, Germany	270805	Ultrasonic bath (DT 31 H. BANDELIN. Berlin, Germany) + isopropyl alcohol (99.7%. SAV Liquid Production. Flintsbach am Inn, Germany), 2 × 1 min. Dried with an air syringe.	
VarseoSmile triniQ	VST	BEGO Bremer Goldschlägerei Wilh. Herbst, Bremen, Germany	n.a.	Ultrasonic bath (DT 31 H. BANDELIN. Berlin, Germany) + ethanol (96%. Otto Fischar. Saarbrücken, Germany), 3 min precleaning and 2 min final cleaning. Dried with an air syringe.	
VarseoSmile Crown Plus	VSC		601758		Otoflash G171 (NK Optik. Baierbrunn. Germany), 12.5 mW/cm^2^ per flash 360–410 nm, and 2 × 1500 flashes. Nitrogen atmosphere. A total of 1500 flashes each side.

**Table 2:** Descriptive statistics showing the mean and standard deviation of FS, E_calc, and m depending on the tested groups.

		CCR		DCR	BRT	CRT	FCR	VST	VSC	VSC		BRC
		Midas	Pro 55s	Asiga Max						TrixPrint2	Varseo XS	-
FS [MPa] Mean ± SD	Initial	130 ± 16.1 ^AIy^	125 ± 7.26 ^AIz^	122 ± 8.91 ^dIy^	104 ± 12.2 ^bIz^	116 ± 6.97 ^cdIy^	112 ± 10.9 ^bcdIz^	109 ± 8.49 ^bcIz^	77 ± 13.0 ^BaIy^	54 ± 9.17 *^AIy^	74 ± 9.92 ^BIy^	167 ± 15.5 ^IIz^
	24 h, H_2_O	97.4 ± 17.2 ^AIx^	103 ^A^ ± 6.14 ^AIy^	120 ± 8.86 ^dIy^	89.6 ± 11.8 ^bIy^	106 ± 12.6 *^cIx^	95.9 ± 10.0 ^bcIy^	84.7 ± 11.3 ^bIy^	58.0 ± 8.3 ^BaIx^	46.3 ± 10.0 *^AIxy^	57.6 ± 8.56 ^BIx^	151 ± 8.68 ^IIy^
	10,000 TC	84.5 ± 11.4 ^BIx^	74.0 ± 7.94 ^AIx^	112 ± 7.62 ^cIIx^	74.3 ± 13.2 ^bIx^	106 ± 8.62 ^cIIx^	83.4 ± 14.2 ^bIx^	76.3 ± 7.54 ^bIx^	58.5 ± 9.15 ^BaIx^	38.6 ± 9.59 ^AIx^	55.8 ± 13.8 *^BIx^	124 ± 11.7 ^IIx^
E_calc [GPa] Mean ± SD	Initial	5.35 ± 0.63 ^BIIx^	3.99 ± 0.38 ^AIx^	3.03 ± 0.33 ^cIx^	2.32 ± 0.11 *^abIx^	2.43 ± 0.26 ^abIx^	2.63 ± 0.23 ^bIy^	2.54 ± 0.46 ^abIy^	2.23 ± 0.15 ^BaIx^	1.93 ± 0.13 ^AIx^	1.99 ± 0.17 ^AIx^	5.89 ± 1.06 ^IIx^
	24 h, H_2_O	5.73 ± 0.50 ^BIIx^	4.06 ± 0.34 *^AIx^	3.32 ± 0.33 ^cIx^	2.61 ± 0.20 ^bIy^	2.69 ± 0.37 *^bIy^	2.29 ± 0.17 ^aIx^	2.03 ± 0.10 ^aIx^	2.28 ± 0.15 *^BaIx^	2.11 ± 0.12 ^AIy^	2.22 ± 0.26 ^ABIy^	5.67 ± 0.94 ^IIx^
	10,000 TC	5.33 ± 0.52 ^BIIx^	4.02 ± 0.27 ^AIx^	3.29 ± 0.50 ^cIx^	2.57 ± 0.12 ^bIy^	2.53 ± 0.27 ^bIxy^	2.44 ± 0.21 ^bIx^	1.91 ± 0.10 ^aIx^	2.36 ± 0.16 ^BbIx^	2.40 ± 0.24 ^BIz^	2.12 ± 0.20 ^AIxy^	5.22 ± 0.63 ^IIx^
m (95%CI)	Initial	9.5 (5; 16)	19.9 (12; 33)	15.8 (9; 26)	9.6 (5; 16)	19.7 (11; 33)	11.8 (7; 20)	15.0 (9; 25)	6.6 (4; 11)	7.9 (4; 14)	8.6 (5; 15)	12.3 (7; 21)
	24 h, H_2_O	6.8 (3; 11)	20.7 (13; 24)	15.8 (9; 27)	8.7 (5; 15)	9.4 (5; 16)	11.2 (6; 19)	8.4 (5; 14)	8.3 (5; 14)	5.6 (3; 10)	7.8 (4; 13)	20.2 (12; 33)
	10,000 TC	8.9 (5; 14)	10.7 (6; 18)	18.0 (10; 30)	6.4 (3; 11)	14.4 (8; 24)	6.9 (4; 12)	11.6 (7; 20)	7.6 (4; 13)	5.0 (3; 9)	5.6 (3; 10)	12.9 (7; 22)

* indicates deviation from a normal distribution. ^AB^ different uppercase letters indicate significant differences between the same material printed on different printers. ^abcd^ different lowercase letters indicate significant differences between different materials printed on the same printer. ^I II^ different roman numerals indicate significant differences between tested groups and the control group. ^xyz^ different lowercase letters indicate significant differences between different aging states.

**Table 3:** Descriptive statistics showing the mean and standard deviation of HM and EIT depending on the tested groups.

		CCR		DCR	BRT	CRT	FCR	VST	VSC	VSC		BRC
		Midas	Pro 55s	Asiga Max						TrixPrint2	Varseo XS	-
HM [MPa] Mean ± SD	Initial	241 ± 345.0 ^AIx^	224 ± 38.4 ^AIx^	230 ± 23.6 ^dIy^	152 ± 13.0 ^abcIx^	170 ± 18.5 ^cIx^	156 ± 17.9 ^bcIx^	135 ± 12.4 ^aIx^	148 ± 12.2 ^ABabIx^	152 ± 10.8 ^BIx^	138 ± 13.0 ^AIx^	453 ± 34.8 ^IIy^
	24 h, H_2_O	241 ± 26.2 ^AIx^	226 ± 34.9 ^AIx^	216 ± 20.6 ^dIxy^	155 ± 11.9 ^bcIx^	173 ± 16.5 ^cIx^	173 ± 20.7 ^abIy^	138 ± 11.8 ^aIx^	153 ± 16.2 ^BbIx^	157 ± 10.4 *^BIx^	129 ± 24.7 ^AIx^	438 ± 20.7 ^IIy^
	10,000 TC	245 ± 36.7 ^BIx^	211 ± 24.8 *^AIx^	210 ± 17.8 ^cIx^	158 ± 11.4 ^bIx^	167 ± 11.8 ^bIx^	152 ± 14.9 ^bIx^	125 ± 19.9 *^aIx^	157 ± 16.2 *^BbIx^	161 ± 16.1 ^BIx^	139 ± 17.8 ^AIx^	394 ± 17.6 ^IIx^
EIT [GPa] Mean ± SD	Initial	7.40 ± 1.40 ^AIx^	6.63 ± 1.73 ^AIx^	5.74 ± 0.66 ^cIx^	4.37 ± 0.39 ^bIx^	4.15 ± 0.46 ^abIx^	4.33 ± 0.39 ^bIy^	3.65 ± 0.28 ^aIy^	4.21 ± 0.43 *^BbIx^	4.16 ± 0.33 *^BIx^	3.70 ± 0.44 ^AIx^	11.9 ± 1.02 ^IIy^
	24 h, H_2_O	7.46 ± 0.86 ^BIx^	6.71 ± 1.47 ^AIx^	5.40 ± 0.62 ^cIx^	4.34 ± 0.31 ^bIx^	4.20 ± 0.59 ^bIx^	3.88 ± 0.52 ^abIx^	3.54 ± 0.25 ^aIxy^	4.37 ± 0.40 ^BbIx^	4.34 ± 0.22 ^BIx^	3.28 ± 0.75 ^AIx^	11.5 ± 0.47 ^IIy^
	10,000 TC	7.35 ± 1.58 ^BIx^	6.01 ± 1.19 ^AIx^	5.62 ± 0.52 ^dIx^	4.49 ± 0.27 ^cIx^	3.94 ± 0.51 ^bIx^	4.00 ± 0.52 ^bcIxy^	3.30 ± 0.50 *^aIx^	4.39 ± 0.37 ^BbcIx^	4.28 ± 0.40 *^BIx^	3.47 ± 0.62 ^AIx^	10.6 ± 0.62 ^IIx^

* indicates deviation from a normal distribution. ^AB^ different uppercase letters indicate significant differences between the same material printed on different printers. ^abcd^ different lowercase letters indicate significant differences between different materials printed on the same printer. ^I II^ different roman numerals indicate significant differences between tested groups and the control group. ^xyz^ different lowercase letters indicate significant differences between different aging states.

**Table 4:** Descriptive statistics showing the minimum/median/maximum of E_RFDA, G_RFDA, and ν depending on the tested groups.

		CCR		DCR	BRT	CRT	FCR	VST	VSC	VSC		BRC
		Midas	Pro 55s	Asiga Max						TrixPrint2	Varseo XS	-
E_RFDA [GPa] Min/Med/Max 95%CI	Initial	-	9.68/9.89/9.92 *^Ix^	7.79/7.80/7.80 *^fIy^	5.69/5.70/5.71 ^dIy^	5.72/5.73/5.74 ^eIz^	5.34/5.34/5.34 *^cIx^	4.05/4.05/4.05 *^aIy^	4.44/4.44/4.44 ^AbIx^	4.63/4.63/4.63 *^BIy^	4.86/4.87/4.88 ^CIy^	14.73/14.73/14.80 *^IIz^
	24 h, H_2_O	-	10.2/10.2/10.2 ^Iz^	7.76/7.77/7.79 ^fIx^	5.86/5.87/5.88 ^eIz^	5.66/5.66/5.67 *^dIy^	5.45/5.46/5.46 ^cIz^	4.06/4.07/4.07 ^aIz^	4.91/4.91/4.92 *^BbIz^	4.88/4.89/4.89 ^AIz^	5.42/5.42/5.42 ^CIz^	14.5/14.5/14.6 ^IIy^
	10,000 TC	-	10.0/10.1/10.1 ^Iy^	7.66/7.79/7.80 *^fIx^	5.52/5.53/5.53 *^aIx^	5.56/5.57/5.57 *^eIx^	5.44/5.44/5.44 ^dIy^	4.04/4.04/4.04 *^bIx^	4.47/4.47/4.47 ^CcIy^	4.37/4.37/4.37 ^Bix^	4.10/4.10/4.10 ^Aix^	14.0/14.0/14.0 ^IIx^
G_RFDA [GPa] Min/Med/Max 95%CI	Initial	-	3.76/3.76/3.76 *^Ix^	2.97/2.97/2.97 *^fIz^	2.06/2.06/2.07 *^dIy^	2.08/2.09/2.09 *^eIy^	1.94/1.95/1.95 *^cIx^	1.45/1.46/1.46 *^aIy^	1.87/1.87/1.88 *^BbIy^	1.85/1.85/1.85 *^AIy^	1.89/1.89/1.89 ^CIy^	5.74/5.74/5.75 *^IIz^
	24 h, H_2_O	-	3.90/3.90/3.90 ^Iy^	2.95/2.96/2.96 *^fIy^	2.14/2.15/2.15 ^eIz^	2.10/2.11/2.11 ^dIz^	1.99/1.99/1.99 ^cIz^	1.39/1.42/1.44 ^aIx^	1.87/1.87/1.87 ^AbIy^	1.87/1.87/1.87 ^AIz^	1.89/1.90/1.90 *^BIz^	5.64/5.64/5.64 ^IIy^
	10,000 TC	-	3.89/3.90/3.91 ^Iy^	2.90/2.91/2.93 ^fIx^	2.01/2.01/2.01 ^dIx^	2.02/2.02/2.03 *^eIx^	1.97/1.97/1.97 ^cIy^	1.52/1.52/1.52 *^aIz^	1.68/1.68/1.68 *^AbIx^	1.74/1.74/1.74 *^Bix^	1.80/1.80/1.80 *^CIx^	5.44/5.44/5.45 *^IIx^
ν	Initial	-	0.290/0.316/0.320 *^Iy^	-/0.313/- *^bIx^	-/0.381/- *^dIz^	0.370/0.373/0.380 ^cIy^	-/0.372/- *^cIx^	-/0.392/- ^eIy^	-/0.186/- *^BaIx^	-/0.176/- ^Aix^	-/0.286/- ^CIy^	0.280/0.282/0.290 *^IIx^
	24 h, H_2_O	-	0.300/0.306/0.310 ^Iy^	0.310/0.314/0.320 ^aIy^	-/0.368/- ^cIx^	-/0.345/- ^bIx^	-/0.372/- ^dIx^	0.410/0.439/0.460 ^eIz^	0.310/0.313/0.320 ^BaIy^	0.300/0.308/0.310 ^AIz^	-/0.427/- *^CIz^	-/0.287/- ^IIy^
	10,000 TC	-	0.280/0.289/0.300 ^x^	0.320/0.331/0.340 ^bIz^	-/0.374/- ^cIy^	-/0.375/- ^dIz^	-/0.382/- *^eIy^	-/0.327/- * ^aIx^	0.330/0.330/0.330 ^CbIz^	-/0.256/- ^BIy^	-/0.142/- ^Aix^	-/0.289/- *^IIz^

* indicates deviation from a normal distribution. ^AB^ different uppercase letters indicate significant differences between the same material printed on different printers. ^abcdef^ different lowercase letters indicate significant differences between different materials printed on the same printer. ^I II^ different roman numerals indicate significant differences between tested groups and the control group. ^xyz^ different lowercase letters indicate significant differences between different aging states.

## Data Availability

The raw data supporting the conclusions of this article will be made available by the authors on request.
